# Total Thyroidectomy with Harmonic Scalpel Combined with Gelatin Thrombin Hemostatic: A Focus on the Elderly Population—A Multicentric Study

**DOI:** 10.3390/medicina61030496

**Published:** 2025-03-13

**Authors:** Simona Parisi, Claudio Gambardella, Roberto Ruggiero, Giovanni Docimo, Vincenzo Marotta, Adelmo Gubitosi, Federico Maria Mongardini, Valerio D’Orazi, Francesca Fisone, Luigi Brusciano, Salvatore Tolone, Ludovico Docimo, Francesco Saverio Lucido

**Affiliations:** 1 Division of General, Oncological, Mini-Invasive and Obesity Surgery, University of Study of Campania “Luigi Vanvitelli”, via Luigi Pansini n° 5, 80131 Naples, Italyroberto.ruggiero@unicampania.it (R.R.); adelmo.gubitosi@unicampania.it (A.G.); f.mongardini@gmail.com (F.M.M.); francesca.fisone@unicampania.it (F.F.); luigi.brusciano@unicampania.it (L.B.); salvatore.tolone@unicampania.it (S.T.); ludovico.docimo73@gmail.com (L.D.); francescosaverio.lucido@unicampania.it (F.S.L.); 2 Division of Thyroid Surgery, University of Study of Campania “Luigi Vanvitelli”, 80131 Naples, Italy; giovanni.docimo@unicampania.it; 3 Division of Endocrinology and Diabetology, AOU San Giovanni di Dio e Ruggi d’Aragona, 84131 Salerno, Italy; vincenzo.marotta@sangiovannieruggi.it; 4 Division of Surgery, Section of Endocrine Surgery and Diabetic Foot Surgery, “Fabia Mater” Hospital, 00171 Rome, Italy; valerio.dorazi@uniroma1.it; 5 Department of Surgery, Sapienza University of Rome, 00185 Rome, Italy

**Keywords:** total thyroidectomy, elderly, harmonic scalpel, gelatin–thrombin matrix, Floseal

## Abstract

*Background and Objectives*: With the increasing life expectancy, the frequency of total thyroidectomies in elderly patients has risen, raising concerns regarding hemorrhage and recurrent laryngeal nerve palsy compared to the general population. Therefore, considering the frequent alteration of the coagulation status in such patients, innovative methods able to reach an accurate hemostasis appear highly desirable. This retrospective multicentric study aimed to compare the postoperative outcomes of patients treated with conventional hemostasis with patients treated with the Harmonic Scalpel (HS) and gelatin–thrombin matrix (Floseal). *Materials and Methods*: Patients undergoing total thyroidectomy were retrospectively enrolled and categorized into two groups: Group A patients underwent surgery with the Harmonic Scalpel and Floseal, while Group B underwent traditional hemostasis surgery with ligations and monopolar electrocautery. The primary endpoint was the drain output after 24 and 48 h and the presence of significant blood loss. Secondary endpoints included the presence of seroma, wound infection, hematoma, laryngeal nerve palsy, surgery duration, and onset of post-surgical hypocalcemia. *Results*: From January 2014 to January 2024, 870 individuals participated in the study. Group A (gelatin–thrombin and HS) comprised 502 patients, while Group B (Standard Hemostasis—control group) comprised 368 patients. The 24 h drain output was 52 ± 25 mL in Group A vs. 113 ± 27 mL in Group B, *p* = 0.003, while the 48 h drain output was 95 ± 29 mL in Group A and 113 ± 27 mL in Group B (*p* = 0.002). Significant blood loss occurred in eight patients (2.2%) of Group B vs. three cases (0.6%) in Group A (*p* = 0.039). Also, neck hematoma (*p* = 0.012), seroma (*p* = 0.005), and reoperation (*p* = 0.052) values were significantly lower in Group A. *Conclusions*: Surgery aided with HS, and gelatin–thrombin was associated with lower major and minor complications compared to the conventional approach, guarantying reduced operative time, ensuring hemostasis, and preserving parathyroid glands, even in elderly patients.

## 1. Introduction

Total thyroidectomy (TT) is a common procedure in endocrine surgery, indicated for several thyroid disorders, including cancer, hyperthyroidism, and goiter [1]. The thyroid gland’s rich vascularity emphasizes the critical role of hemostasis in ensuring a successful surgery attempt and, at the same time, minimizing the risk of structural damage (i.e., recurrent laryngeal nerves and parathyroid glands) [2].

Traditional methods for achieving hemostasis involve the clamp, tie, and cut technique, with or without electrocautery. However, these methods, especially monopolar cautery, pose risks due to heat dispersion and imprecise coagulation [3]. Innovative vessel sealing devices, such as the Harmonic Scalpel (HS) (Focus Shears, Ethicon, Raritan, NJ, USA), have been introduced to improve bleeding control, reduce operative time, and minimize postoperative complications [4].

Moreover, several topical hemostatic agents (i.e., gelatin sponges, fibrin sealants, microfibrillar collagen, and hemostatic matrices) have been proposed in thyroid surgery to complete and optimize the hemostasis, attempting to also reduce complications such as postoperative seroma [5]. Floseal Hemostatic Matrix^®^ (Baxter SpA, Rome, Italy) is an efficient topical hemostatic agent containing gelatin–thrombin designed to reduce blood loss in various surgical procedures, including thyroid surgery [6]. In a previous study, a combination of HS and gelatin–thrombin has shown effectiveness in reducing post-surgical drain output when tested in a general population of patients affected by thyroidal disease.

With an increase in life expectancy, surgical interventions for thyroid disorders in the elderly have become more common. The term “elderly” lacks a clear definition in the literature; however, studies have shown that the increase in mortality with old age depends more on biological age than chronological age and on the number of associated comorbidities [7].

This study aims to analyze and compare the outcomes of traditional TT approaches versus the combination of HS and gelatin–thrombin in elderly patients, focusing on operative time, complications, and postoperative outcomes.

## 2. Materials and Methods

### 2.1. Study Design

This study adheres to the STROBE statement for cohort studies, providing comprehensive guidelines for reporting observational studies [8]. It is a retrospective multicentric study aiming to compare the effectiveness of the combined use of gelatin–thrombin and HS with traditional hemostatic procedures (gauze, ligature, and electrocauterization) during total thyroidectomy (TT) in elderly patients. The study was conducted in accordance with the ethical principles outlined in the Declaration of Helsinki, and written informed consent was obtained from all subjects.

### 2.2. Study Setting and Study Population

Patients with thyroidal disease who were referred to the General Surgery Unit of Campania University “Vanvitelli” (Naples, Italy), Division of Surgery-Section of Endocrine Surgery and Diabetic Foot Surgery “Fabia Mater” Hospital (Rome, Italy), and the Department of Surgery “Sapienza” University (Rome, Italy) from January 2014 to January 2024 were considered in the study. Inclusion criteria were subjects with an indication for total thyroidectomy and age ≥65 years. Exclusion criteria included diabetes, metabolic and chronic renal disease, previous neck irradiation or surgery, cervicomediastinal goiters, known coagulopathy, active or a history of malignant systemic disease, and drug or alcohol abuse.

Patients affected by proliferative hemopoietic disorders, heart failure, and the use of an anticoagulant drug were also excluded from the study because these conditions could represent a bias when verifying the effectiveness of the hemostat. Moreover, patients who were enrolled in the control group, who were not treated with hemostatic agents, but who received them following the development of complications, were also excluded from the study. Additionally, candidates for video-assisted thyroidectomy (minimally invasive), pregnant or lactating females, and cases requiring central compartment or other lymph node aspirations were excluded.

All patients underwent preoperative blood sampling, a neck ultrasound, fine-needle cytology (FNC) of the thyroid neoplasm, and fiberoptic laryngoscopy tests in the 30 days prior to surgery. After the referral for surgery, each patient received a detailed explanation of the procedure from the medical staff and had to sign a personalized informed consent. All the operations were performed by experienced endocrine surgeons (over 200 total thyroidectomies).

Clinical data were collected in an electronic database and retrospectively analyzed. Patients were divided into two groups: Group A, patients who underwent TT with the hemostatic procedure using gelatin–thrombin and HS, and Group B, patients who underwent TT with traditional hemostatic procedures alone (clamp, tie, and electrocauterization), which was considered the control group.

Preoperative clinical and demographical parameters (age, sex, BMI, thyroid volume, type of intervention, intraoperative blood loss, volume of drainage, and thyroid disease) were evaluated as well as the postoperative outcomes (day of discharge, rates of complications, onset of seroma or hemorrhage, and reoperation). The follow-up consisted of regular clinical and instrumental examinations of the neck until the 28th day after the discharge.

### 2.3. Surgical Techniques

Total thyroidectomy (TT) involved a Kocher incision of approximately 5 cm at the lower neck crease, positioned two fingers above the suprasternal notch, and performed with a cold scalpel. The platysma muscles were divided, and dissection was carried out on the cervical linea alba. The thyroid lobe was dissected progressively, with vessels ligated and sectioned. The lobe was rotated medially under supervision to preserve the recurrent laryngeal nerve, followed by the removal of the thyroid lobe. The same procedure was repeated for the contralateral lobe. After confirming hemostasis, a drain was placed in the thyroid bed. The cervical linea alba and platysma were sutured using absorbable sutures, and the skin was closed with an intracutaneous running suture. A postoperative drain was placed in the thyroid bed and removed after 48 h.

For patients undergoing TT with gelatin–thrombin and HS (Group A), the following was carried out:

- Dissection and ligation of vascular pedicles were performed using the HS;
- Gelatin–thrombin was applied after the thyroidectomy, particularly in the Gruber and Sappey ligaments, with the aim of avoiding electrocautery injuries to recurrent nerves.

For patients undergoing the traditional hemostasis approach (Group B), the following was carried out:

- Dissection and ligation of vascular pedicles were performed using ligatures, sutures, and electrocautery;
- Bleeding not responsive to surgical hemostasis, particularly in the Gruber and Sappey ligaments, was treated solely with electrocautery or ligation. Patients who were enrolled in the control group (A), who were not treated with hemostatic agents, but who received them following the development of complications, were also excluded from the study.

In both groups, the suction drain was removed after 48 h. A neck ultrasonographic evaluation was conducted 48 h after surgery, and all patients followed the same postoperative protocol.

The surgeries were performed by surgeons with at least 10 years of experience. The enrollment of patients in the two groups is a result of the fact that in one center, devices are used, while in the other, they are not.

The surgery was performed using traditional techniques, without the aid of additional devices for identifying the nerve.

Patients who experienced postoperative complications were mostly managed with conservative therapy, while in cases of massive blood loss, surgical interventions were performed to achieve control of the hemorrhage. For each group, procedures were conducted using the same devices selected for the elective surgery.

### 2.4. Outcomes Measure

Preoperative data, including age, gender, ASA (American Society of Anesthesiologists) score, BMI (Body Mass Index), preoperative serum calcium levels, and thyroid pathology, were obtained from medical records. The duration of surgery was extrapolated from surgical reports and recorded in minutes. It was calculated from the initiation of the skin incision to the completion of the intradermic suture. Drain output data were obtained from nurse reports. Measurements were recorded in milliliters (mL) after 24 and 48 h post-surgery. The eventual presence of significant blood loss, defined as bright red and dense drainage output exceeding 150 mL in two hours, was noted in clinical records. Serum calcium levels were measured at 24 and 48 h post-surgery and reported in milligrams per deciliter (mg/dL). Hypocalcemia was defined as a reduction in calcium levels below 8.9 mg/dL. In cases of symptomatic hypocalcemia, intravenous calcium was administered. Post-surgical complications, such as infections and hematoma, were reported in medical records. Clinical examinations were conducted at 24 and 48 h post-surgery by a surgeon.

The postoperative follow-up control included the following:

- A cervical ultrasound, performed using a 7–13 Hz linear probe (Esaote, MyLab™ X5, Genova, Italy) at 48 h and 7 days after surgery to detect potential hematoma or seroma. Sonographers reported the transversal, longitudinal, and cranio-caudal diameters in millimeters in case of detection.
- In the event of dysphonic voice incidence, laryngoscopy was performed before the discharge.
- Clinical examinations after 3 months by an experienced endocrine surgeon.

### 2.5. Study Endpoints

Primary endpoints were the drain output measured in milliliters (mL) after 24 and 48 h and the presence of significant postoperative blood loss, defined as bright red and dense output exceeding 150 mL in two hours. Secondary endpoints were the presence of seroma, wound infection, hematoma, laryngeal nerve palsy, surgery duration, and post-surgical hypocalcemia.

### 2.6. Statistical Analysis

Continuous variables were described as mean and standard deviation, while categorical variables were described as the number of cases. The population was divided into 2 groups, whether or not hemostat and HS were used. Independent samples *t*-tests were performed to compare the continuous variables (age, days of discharge, BMI, thyroid volume in mL, operation time, and drainage volume); a Test of Proportions was applied to the categorical variable of seroma formation. Moreover, Fisher’s exact test was used to analyze gender and malignant and benign diseases. Statistical significance was considered in the case of a *p*-value < 0.05. Statistical analysis was performed with SPSS version 23 (SPSS©, Chicago, IL, USA).

## 3. Results

### 3.1. Study Population

**Figure 1:**
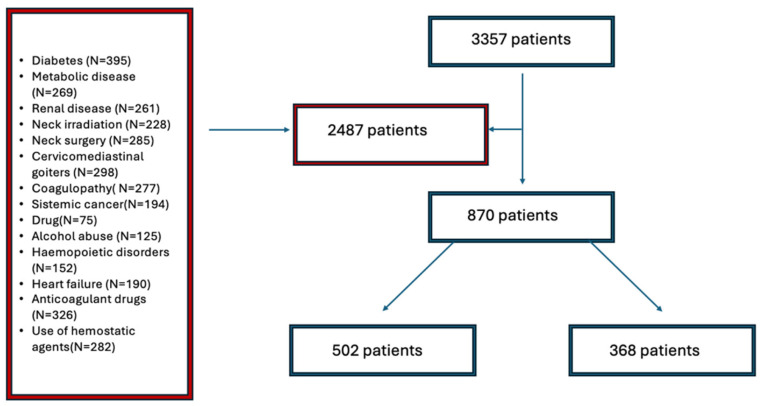
Flow chart.

From January 2014 to January 2024, 3357 patients with thyroidal disease were referred to three high-volume centers. Among them, 2487 patients were excluded, and 870 individuals were enrolled in the study (Figure 1). In Group A (gelatin–thrombin + HS), 502 patients were included while Group B (Standard Hemostasis—control group) was composed of 368 patients. The mean age was 69.2 ± 4.0 years in Group A and 68.8 ± 6.6 years in Group B (*p*-value = 0.099). In total, 626 people were women (74.1%). Demographic and clinical data are detailed in Table 1. No significant differences were observed in other demographic characteristics between the two groups. The primary indication for surgery was a norm functional goiter with compression symptoms in 401 patients. No cases of mortality were observed.

### 3.2. Primary Outcomes

Significant blood loss occurred in eight patients (2.2%) of Group B vs. three cases (0.6%) in Group A (*p* = 0.039). Drain output at 24 h was significantly lower in Group A (52 ± 25 mL vs. 83 ± 28 mL, *p* = 0.002). Similarly, the results were confirmed at 48 h (Group A 95 ± 29 mL vs. Group B 113 ± 27 mL, *p* = 0.003) [Table 2].

### 3.3. HS (Harmonic Scalpel), TT (Total Thyroidectomy), and mL (Milliliters)

Regarding the secondary outcomes, 28 participants experienced a seroma, 9 (1.9%) in Group vs. 19 (5.1%) in Group B, with a statistically significant difference (*p* = 0.005) [Table 2]. Twenty-two patients presented neck hematoma: seven participants (1.4%) in Group A vs. fifteen (4.3%) in Group B (*p* = 0.012). A neck hematoma requiring surgical revision occurred in nine patients: two included in Group A (2.4%) and seven (1.9%) in Group B (*p* = 0.030). The other cases were all conservatively managed with simple aspiration [Table 2].

No cases of wound infections were reported.

Post-surgical transient hypocalcemia was observed in 38 patients in Group A (7.5%) vs. 42 in Group B (11.4%), with *p* = 0.005. Only two cases of laryngeal nerve paste were reported, one for each group (0.2% vs. 0.30% in Groups A and B, respectively) with a *p*-value that was not statistically significant (*p* = 0.825) The surgical procedure was shorter in Group A (68.4 ± 37.64 vs. 75.88 ± 14.78 min, *p* = 0.031) [Table 3]. Hospitalization was not statistically significantly lower in Group A (3.12 ± 0.55 days vs. 3.24 ± 0.96 days, *p* = 0.079).

Patients who experienced postoperative complications were mostly managed with conservative therapy, while in cases of massive blood loss (two in Group A and seven in Group B), further emergent surgery was necessary to achieve control of the hemorrhage. For each group, procedures were conducted using the same devices selected for the elective surgery, and the ligation of the external carotid was not necessary in any of the cases. Patients who developed hematomas received intravenous therapies based on escin and other anti-edema agents. In cases of hypocalcemia, calcium was administered both intravenously and orally; in cases of infections, antibiotic therapy was provided. Laryngeal nerve palsy cases were treated with rehabilitation therapy and speech therapy, and all were resolved within 2 months.

## 4. Discussion

Thyroid disorders, including multinodular goiter and thyroid cancer, often require surgical intervention. Multinodular goiter is prevalent, with an annual volume increase of approximately 5% in elderly patients presenting with large goiters. Total thyroidectomy (TT) is recommended for cases with compressive symptoms or cancer risk, improving the quality of life, even though the vulnerability of patients can generate fear of operative risks. Thyroid cancer, the most common endocrine malignancy, generally has a good prognosis, but elderly patients may face more aggressive forms [9,10,11]. In the past, this population was often a candidate for sub-therapeutic management, even if this therapeutic option was unjustified for many reasons. Frailty index is suggested to better predict thyroidectomy-specific complications than age alone [12]. Moreover, surgery appears to reduce the likelihood of death from thyroid cancer in the elderly. Considering the improved life expectancy, the number of TTs performed in elderly patients has recently increased. Bleeding is considered one of the most dangerous and life-threatening complications, with reported incidence rates in the literature ranging from 0% to 4%. In cases of massive bleeding, immediate intervention becomes necessary, as postoperative hematoma may lead to airway obstruction through laryngeal compression and impairment of venous and lymphatic drainage [13]. Hemorrhage during thyroid surgery can occur at three specific times: intraoperative hemorrhage, hemorrhage within 24 h from the start of the procedure, and postoperative hemorrhage delayed within 7–10 days after surgery, often attributed to the erosion of a blood vessel [14]. Another notable complication is seroma formation, characterized by the accumulation of acute inflammatory exudate in response to surgical trauma during the extended healing process. Moreover, the development of postoperative complications can have various consequences on the length of hospital stay, as well as on the management of these more fragile patients, resulting in a greater burden on caregivers.

In the study in question, we considered the most frequent complications of thyroid surgery as reported in the published literature.

A meta-analysis reported an increased risk of complications following thyroidectomy in the elderly compared to younger cohorts [12]. Nerve injury and hypoparathyroidism management are challenging in older patients, with potential complications like swallowing difficulties and aspiration. A retrospective review indicated an increased complication rate in people > 80 years old undergoing thyroidectomy for multinodular goiter [15]. Postoperative complications include bleeding, hematoma, hypoparathyroidism leading to hypocalcemia, nerve injury, and infections. Unplanned reoperations mostly occurred before discharge, and mortalities happened after discharge, with superficial surgical site infections occurring later.

In another retrospective review involving 161,534 patients, the most common complications following thyroidectomy were bleeding (96%) and hematoma (68%) [16]. The study analyzed the time course of post-surgical complications after total thyroidectomy (TT), revealing that approximately 63% of unplanned reoperations occurred before discharge, while more than 65% of mortalities occurred after discharge, typically within 7 days. Superficial surgical site infections, generally, manifested later.

Postoperative hypoparathyroidism leading to hypocalcemia is another concern, and it is primarily caused by parathyroid gland deficit, with its incidence ranging from 1.7% to 68% in different series. The implementation of ultrasonic mechanical energy, such as the Harmonic Scalpel (HS), has shown significant benefits in thyroid surgery. Several studies [17,18] have demonstrated that the use of HS is safe and effective. HS, in fact, exhibits limited lateral thermal damage, extending up to 2 mm beyond the tissue grasped within the forceps of the device. This property is crucial in thyroid surgery, ensuring safe vascular ligation with minimal risk of damage to the recurrent laryngeal nerve, the external branch of the superior laryngeal nerve, and the parathyroid glands [19,20].

Studies, including a prospective randomized study and observations by Pelizzo et al. [21] and Luo et al. [22], have confirmed that HS is associated with a lower risk of definitive recurrent laryngeal nerve palsy, intraoperative blood loss, and postoperative bleeding compared to conventional hemostasis. Reducing operative time as much as possible is an effective strategy for limiting complications, especially in elderly patients. Currently, operative duration stands out as a crucial factor in comparative studies, highlighting the tangible advantage of employing specialized devices for hemostasis and dissection over traditional methods. In a prior study, innovative devices, such as Harmonic Scalpel (HS) or LigaSure Precise/Small Jaw, were tested and compared to assess the precision of hemostasis and reduction in operative time for both younger and geriatric patients. The results showed promising outcomes, with no statistically significant differences in postoperative complication rates, including hemorrhage, seroma, hematoma, wound infection, transient or definitive hypocalcemia, and transient or definitive recurrent nerve palsy [19].

A recent study investigated a different advanced hemostasis device, the novel LigaSure Small Jaw, adopted for sutureless thyroidectomy, and compared it to the conventional clamp-and-tie technique in one thousand consecutive patients. The overall complication rates of hematoma, hypocalcemia (temporary/permanent), and nerve (temporary/permanent) palsy were 0.9%, 24.9% (24.6%/0.3%), and 1.7% (1.5%/0.2%), respectively. The LigaSure appeared feasible for sutureless thyroidectomy and obtained better outcomes of postoperative hematoma and hypocalcemia than the clamp-and-tie hemostatic technique [23].

The efficacy of the Floseal matrix hemostatic agent was evaluated by Testini et al. in 2009. The mean operating time was significantly reduced in the Floseal group (105 min) compared to traditional total thyroidectomy (TT) (133 min, *p* = 0.02) and the Tabotamp approach (122 min, *p* = 0.0003). Additionally, wound drain removal occurred earlier with Floseal (*p* = 0.006 vs. surgical; *p* = 0.008 vs. Tabotamp), resulting in a shorter postoperative hospital stay [6].

Other hemostatic techniques are available. Resorbable polysaccharide powder (HaemoCer™ Plus) is a plant-based polymer derived from purified vegetable starch and is characterized by a topical hemostat. During thyroid surgery, HaemoCer™ can be directly applied to the bleeding site or used to pack areas with difficult hemostasis. In a recent study, it appeared to be an effective agent. The patients presented lower drainage output and lower incidence of neck hematoma and seroma compared with the control group [5].

To the best of our knowledge, this is the first study assessing the feasibility of a sutureless approach with HS and gelatin–thrombin in elderly patients compared to the traditional approach involving clamp, tie, and cut procedures.

In our study, the most frequent complication was hypocalcemia, in line with what is reported in the literature. The second most concerning complication was the development of a seroma. In the current study, the patient undergoing sutureless TT aided with gelatin–thrombin had lower significant blood loss (three cases (0.6%) in Group A vs. eight patients (2.2%) in Group B, *p* = 0.039) and lower drainage output at 24 and 48 h (52 ± 25 mL vs. 83 ± 28 mL, *p* = 0.002, and 95 ± 29 mL vs. 113 ± 27 mL, *p* = 0.003). Similarly, reoperation due to postoperative bleeding was significantly lower in Group A (two patients (0.4%) vs. seven patients (1.9%), *p* = 0.03), confirming the hemostatic efficacy of HS combined with gelatin–thrombin. These aspects appeared to be of utmost importance since elderly patients often present comorbidities and preventing the risk of postoperative complications and reoperation is mandatory. Considering the postoperative course, patients in Group A presented significantly lower seroma (9 vs. 19, *p* = 0.005), neck hematoma (7 vs. 15, *p* = 0.012), and complications in general (58 vs. 85, *p* < 0.0001), despite the surgical procedure being statistically significantly shorter in Group A (68.4 ± 37.64 vs. 75.88 ± 14.78, *p* = 0.031).

The management of giant thyroid tumors in patients with multiple comorbidities could also have an increased benefit using HS, as reported in the literature [24].

The protocol we proposed is likely associated with higher instrument costs, but a careful cost analysis may instead demonstrate a reduction in expenses when considering the impact on the reoperation rate and the length of hospital stays.

Furthermore, we believe that the combined technique can also be tested in other groups of patients with hematological and cardiovascular comorbidities and those undergoing anticoagulant therapy. Moreover, the proposed strategy could also be used in emergency surgeries for hemorrhagic complications.

Another very interesting aspect is the impact on the duration of the surgical procedure, which appears to be reduced in the experimental group. This offers advantages in terms of operating room management, personnel costs, and, most importantly, the duration of general anesthesia, thereby further protecting older patients from additional related risks.

The study presents some limitations, such as its retrospective nature and the non-matched enrollment in terms of age and gender, even if in the analysis of the data and demographic characteristics, no statistically significant differences were found regarding baseline features. In addition to comparing two different techniques, it also compares the postoperative results of two different surgeons, but this is common in multicentric studies. Moreover, the devices we tested certainly have a higher cost compared to electrocautery. However, this was not the focus of the current study.

## 5. Conclusions

Thyroid pathology is particularly common in elderly patients, given the increased incidence of malignancy and large goiter. However, older age can worsen the postoperative course of patients undergoing TT. In the current series, surgery aided with HS and gelatin–thrombin was associated with lower major and minor complications compared to the conventional approach, guaranteeing reduced operative times, ensuring hemostasis, and preserving parathyroid glands, even in elderly patients. Further larger comparative studies are needed to address this issue.

## Figures and Tables

**Table 1:** The baseline clinical and demographic features in both groups. ° Median and standard deviation.

	Group A (n502) Floseal + HS TT	Group B (n368) Standard TT	*p*-Value
Age (years) °	69.2 ± 4.0	68.8 ± 6.6	0.099
Gender (%)			
-Male	149 (29.7%)	95 (25.9%)	0.176
-Female	353 (70.3%)	273 (74.1%)	0.184
ASA I-II	450 (89.8%)	338 (92.1%)	0.192
ASA III-IV	52 (10.2%)	30 (7.9%)	0.121
BMI (kg/m^2^)	23.3 ± 0.6	22.5 ± 0.5	0.165
Thyroidal disease			
-NORMOFUNCTIONAL NODULES	242 (48.3%)	169 (45.9%)	0.823
Multinodular goiter	117 (23.3%)	83 (22.5%)	0.794
Uninodular goiter	87 (17.3%)	65 (17.7%)	0.898
Follicular adenoma	32 (6.4%)	18 (4.9%)	0.353
Hurthle cell adenoma	6 (1.3%)	3 (0.8)	0.584
-HYPERTHYROID GOITER	176 (35.0%)	138 (37.5%)	0.789
Basedow disease	132 (26.3%)	104 (28.3%)	0.519
Plummer adenoma	44 (8.8%)	34 (9.2%)	0.808
-CARCINOMA			
Papillary	84 (16.7%)	71 (19.1%)	0.768
Follicular	58 (11.5%)	50 (13.6%)	0.368
Medullary	18 (3.6%)	14 (3.8%)	0.865
Anaplastic	8 (1.6%)	7 (1.7%)	0.729
	0	0	-
Preoperative calcemia (mg/dL) °	9.2 ± 0.8	9.3 ± 0.6	0.234
Hypertension	137 (27.3%)	107 (29.0%)	0.562
Smoking	125 (24.9%)	90 (24.5%)	0.880
Cerebrovascular disease	3 (0.6%)	4 (1%)	0.434
Heart ischemic attack	5 (1.0%)	2 (0.5%)	0.460

BMI, Body Mass Index; ASA, American Society of Anesthesiologists.

**Table 2:** Postoperative outcomes in Groups A and B. ° Median and standard deviation.

	Group A (n502) Floseal + HS TT	Group B (n368) Standard TT	*p*-Value
Drain output after 24 h (mL) °	52 ± 25	83 ± 28	0.002 *
Drain output after 48 h (mL) °	95 ± 29	113 ± 27	0.003 *
Significant blood loss (%)	3 (0.6%)	8 (2.2%)	0.039 *
Reoperation	2 (0.4%)	7 (1.9%)	0.030 *
Day of discharge	3.21 ± 0.75	3.48 ± 0.89	0.091
Seroma	9 (1.9%)	19 (5.1%)	0.005 *
Surgical site infection	0	0	-
Neck hematoma	7 (1.4%)	15 (4.3%)	0.012 *
Laryngeal nerve palsy	1 (0.2%)	1 (0.3%)	0.825
Mortality	0	0	-
Post-surgical hypocalcemia (%)	38 (7.5%)	42 (11.4%)	0.052

* Statistical significant.

**Table 3:** Intraoperative outcomes in Groups A and B. ° Median and standard deviation.

	Group A (n502) Floseal + HS TT	Group B (n368) Standard TT	*p*-Value
Operation Time (min) °	68.4 ± 37.64	75.88 ± 14.78	0.031 *
Thyroid Volume (mL) °	29.97 ± 7.45	32.01 ± 3.45	0.119
Definitive Pathology			
Malignant Disease	199 (39.5%)	134 (36.4%)	
Benign Disease	303 (60.5%)	234 (63.6%)	0.677

* Statistical significant.

## Data Availability

The datasets used and/or analyzed during the current study are available from the corresponding author upon reasonable request.
